# Comparison of Different Aerogel Granules for Use as Aggregate in Concrete

**DOI:** 10.3390/gels9050406

**Published:** 2023-05-12

**Authors:** Torsten Welsch, Yannick Vievers, Martina Schnellenbach-Held, Danny Bialuschewski, Barbara Milow

**Affiliations:** 1 Institute for Structural Concrete (ISC), University of Duisburg-Essen, 45141 Essen, Germany; yannick.vievers@uni-due.de (Y.V.); massivbau@uni-due.de (M.S.-H.); 2 Institute of Inorganic Chemistry, Nanostructured Cellular Materials, University of Cologne (UoC), 50939 Cologne, Germany; danny.bialuschewski@uni-koeln.de (D.B.); barbara.milow@dlr.de (B.M.); 3 German Aerospace Center (DLR), Institute of Materials Research, Aerogels and Aerogel Composites, 51147 Cologne, Germany

**Keywords:** silica aerogel, aerogel granule, aerogel concrete, lightweight aggregate concrete, compression strength, thermal insulation

## Abstract

In previous work of this group, a structural lightweight concrete was developed by embedding silica aerogel granules in a high-strength cement matrix. This concrete, called high-performance aerogel concrete (HPAC), is a lightweight building material characterized by its simultaneous high compressive strength and very low thermal conductivity. Besides these features, high sound absorption, diffusion permeability, water repellence and fire resistance qualify HPAC as an interesting material for the construction of single-leaf exterior walls without any further insulation. During the development of HPAC, the type of silica aerogel was found to majorly influence both fresh and hardened concrete properties. To clarify these effects, a systematic comparison of SiO_2_ aerogel granules with different levels of hydrophobicity as well as different synthesis methods was conducted in the present study. The granules were analyzed for their chemical and physical properties as well as their compatibility in HPAC mixtures. These experiments included determinations of pore size distribution, thermal stability, porosity, specific surface and hydrophobicity, as well as fresh/hardened concrete experiments such as measurements of compressive strength, flexural bending strength, thermal conductivity and shrinking behavior. It was found that the type of aerogel has a major influence on the fresh and hardened concrete properties of HPAC, particularly compressive strength and shrinkage behavior, whereas the effect on thermal conductivity is not very pronounced.

## 1. Introduction

The construction industry is facing challenges resulting from climate change and political decisions in this context, such as increased demands on building insulation. In many countries of the northern hemisphere, solutions such as external thermal insulation systems with layers of different materials are state-of-the-art. However, these solutions are the object of criticism due to several aspects such as flammability, recyclability, growth of algae, limited architectural freedom and so on. One approach to the solution of these challenges is the so-called high-performance aerogel concrete (HPAC), which consists of silica aerogels embedded in a high-strength cement matrix. HPAC allows for concrete constructions combining load carrying and insulating properties without any additional insulation layers. Besides a remarkable relation between compressive strength and thermal conductivity, HPAC is characterized by high fire resistance, high acoustic insulation, low density, recyclability and free formability, which are highly desirable in conventional constructions as well as 3D printing [1,2,3,4]. General suitability for use in reinforced construction members has also been shown [5].

**Figure 1:**
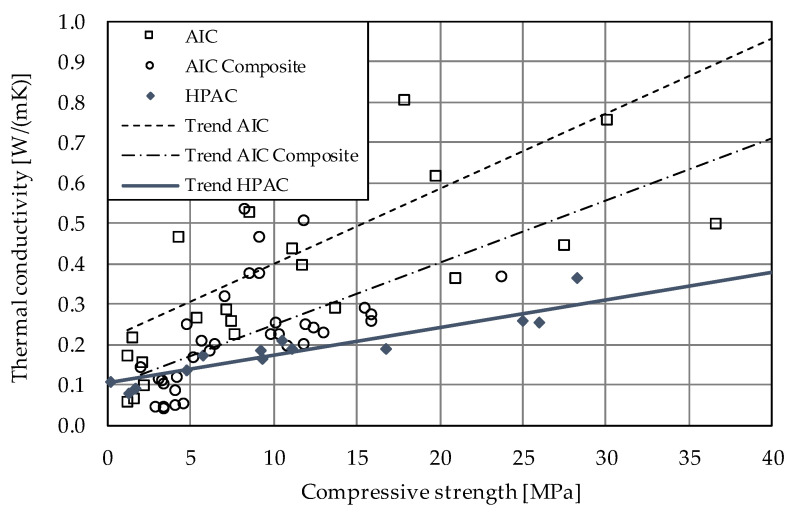
Relation between compressive strength and thermal conductivity of aerogel (incorporated) concretes (AICs), aerogel composite concretes (AIC-composites) and high-performance aerogel concrete (HPAC) (Reprinted/adapted with permission from Ref. [5], 2022, John Wiley and Sons).

Since the first attempts to develop aerogel concrete in 2008 [6], the material has attracted attention worldwide, with the aim of improving the relation of compressive strength and thermal conductivity in order to make aerogel concrete ready for use in building practice. A recent overview of the work on aerogel concretes of the last decade can be found in [5]; the results of this study in terms of compressive strength and thermal conductivity are summarized in Figure 1. From this comparison, the differences between aerogel (incorporated) concretes (AICs), aerogel incorporated concrete composite (AIC-composite) and high-performance aerogel concrete (HPAC) become obvious. Besides aspects resulting from concrete technology such as type of binder, water–cement ratio, additives and so on, the type of silica aerogel was found to have an impact on concrete properties. Most researchers used hydrophobic silica aerogel granules for the following reasons: First, the composite should consist of purely mineral materials to provide fire resistance and simple recyclability. Second, water uptake by the aerogel particles should be avoided to preserve their insulating properties. The hydrophobicity of an aerogel is commonly achieved by adding a thin film on the (inner) surface of the aerogel of a hydrophobic agent. These agents usually consist of long non-polar alkyl chains with a carboxyl-group that forms a chemical bond with the silica surface. However, the hydrophobic surface of the particles tends to interfere with the bond between the aerogel and the cement matrix. It is assumed that the formation of an increased amount of air voids and micro-cracks in the contact zone between the particles and the matrix leads to a reduction in the compressive strength, which is actually worsened by the strong tendency of aerogel concretes to shrink [7]. This typical phenomenon in aerogel concretes is caused by finely ground cements and additives, low water–cement ratios, and the absence of any stiffening aggregate skeleton. Shrinking strains up to 12‰ were found to be typical for (high-performance) aerogel concretes, which is app. 20 times higher than the shrinkage deformation of normal concrete. These high shrinkage strains not only interfere with the accuracy of proper sizing of construction members, but also enhance the propagation of micro-cracks, resulting in lower compressive and tensile strength. Other consequences of enhanced shrinkage affect the production of reinforced construction members free of macro-cracks [5].

The influence of the hydrophobicity of aerogel granules on fresh and hardened concrete properties has not yet been systematically investigated and is therefore not sufficiently understood. The aim of the research presented in this study was to contribute to a better understanding of the relation between surface chemistry and concrete properties via a comparison of different aerogels. For this purpose, silica aerogels from different producers and with different hydrophobicity values were characterized intensively, as is subsequently presented. The mechanical properties of these aerogels are largely comparable with each other and are not considered to contribute to the strength of the composite: the compression strength of silica aerogels with the densities considered in this study is about a decimal power less in comparison with the strength of cement matrix [8]. Thus, stresses on aerogel concretes are supposed to be mainly carried by the cement matrix. However, to evaluate the possible effects of the different mechanical properties of aerogels, one granule type that exhibited a much higher compressive strength and compression modulus was involved. As it had high hydrophobicity, this enabled a direct comparison with a granule type from a different producer with a similarly high hydrophobicity but much lower compressive strength and bulk modulus.

## 2. Materials and Methods

### 2.1. Materials

As mentioned before, aerogels with different hydrophobicity values and different mechanical properties were chosen for this study. From Table 1 it can be seen that KEEY Aerogel provided granules with different rates of hydrophobicity, labeled SICLA X%, and a pre-commercial product, labeled SICLA Lite. SICLA X% aerogels were produced with the same synthesis method using sodium silicate as the silica precursor and exhibited identical mechanical properties, but hydrophobicity was adjusted during the surface modification process by using different amounts of hydrophobic agent, noted as %. SICLA Lite granules were produced through a different synthesis method. The aerogel SUFA shows high hydrophobicity as well as high compressive strength and compression modulus. It was chosen to evaluate possible effects from different mechanical properties and synthesized similarly to flexible hybrid silica aerogels from already hydrophobic precursors [9]. Two types of granules from Cabot (P100 and P250F) were involved due to their high market penetration, serving as a reference for this study. To enable a comparison with hydrophilic granules, the Cabot P100 was pyrolyzed to remove any hydrophobic agent. The obtained material was labeled P100PYR. For comparison reasons, another commercially available granule type, Enersens Kwark, was included.

To compare the effects of different aerogel granules on fresh and hardened concrete properties, a reference concrete mixture, called HPAC_50-P100, served as a base. As can be seen from the mixture composition given in Table 2, this reference mixture contains 46.39 vol% of Cabot P100 aerogel [10]. For the comparison of the aerogels, Cabot P100 was replaced by the different aerogels 1:1, and the other components were kept constant. However, since the hydrophobicity of each granule type varies, the water content of the reference mixture had to be adjusted: to achieve a consistency similar to that of the reference mixture, the water was added gradually during the mixing process. A low water–cement ratio is generally desirable to increase compressive strength, provided that the workability of the fresh concrete is still adequate. The cement used in this study was ordinary Portland cement (OPC) CEM I 52.5 R. A silica suspension, a polycarboxylate ether-based superplasticizer and a stabilizer were added to the mixture as concrete additives. These additives were kept unaltered for the investigations of the different aerogel types, with the exception of Kwark, as preliminary tentative tests for the mixing of HPAC containing Kwark revealed that no workable mixtures could be obtained. After different investigations of this mixture, a chemical incompatibility between the aerogel particles and the superplasticizer was found to cause this behavior. Replacing the superplasticizer with a different PCE-based additive solved this issue.

### 2.2. Methods for the Characterization of Aerogels

For a full understanding of aerogel behavior, thorough characterization is recommended. Therefore, a basic study was carried out for the different silica aerogels used in the HPAC composites.

The thermal conductivity was measured using the heat flow meter apparatus HFM 436 Lambda from NETZSCH (Selb, Germany). The thermal conductivity of the aerogel granular material was measured using a box with a height of 20 mm and a ground 300 mm × 300 mm following EN 12664:2001 [11].

Thermogravimetric analyses were performed using a Thermo-Microbalance TG 209 F1 Iris from NETZSCH (Selb, Germany). All experiments were performed under the same conditions with a constant gas flow of 20 or 40 mL/min, a temperature gradient of 10 K/min and a temperature range from 25 to 800 °C. Afterwards, measurements were performed both under inert nitrogen atmosphere and under oxidative conditions with synthetic air (20/80 *v*/*v* O_2_/N_2_).

Pyrolization of the sample P100PYR was performed at 700 °C with a heat ramp of 400 °C/h for 3 h in a F 150-500/13 oven from Gero Carbolite (Neuhausen, Germany).

The skeletal density was measured with an AccuPyc^®^ II 1340 from Micromeritics™ (Unterschleissheim, Germany). The skeletal density was calculated by

$$\rho_{skeletal}=\frac{m_{sample}}{V_{solid\;backbone}}\;\mathrm{in}\;[g/{\mathrm{cm}}^{3}].$$

The enveloping density of an aerogel body is defined by the ratio of its mass to its volume. The measurements of the irregularly shaped porous aerogel granulate samples were determined using a GeoPyc^®^ 1360 Envelope Density Analyzer from Micromeritics™ (Unterschleissheim, Germany). The envelope density was calculated by

$$\rho_{envelope}=\frac{m_{sample}}{V_{sample}}\;\mathrm{in}\;[g/{\mathrm{cm}}^{3}].$$

The apparent density (also known as bulk density) was determined according to ASTM D2854 [12]. It was ascertained by weighing aerogel granules in bulk and measuring the volume in a measuring cylinder. It was calculated by

$$\rho_{apparent}=\frac{m_{granules\;bulk}}{V_{fill\;level}}\;\mathrm{in}\;[g/{\mathrm{cm}}^{3}].$$

With known skeletal and envelope density, the porosity was calculated by

$$\phi =1-\frac{\rho_{skeletal}}{\rho_{envelope}}.$$

Sorption methods were performed for the determination of the inner surface areas using a TriStar II 3020 from Micromeritics™ (Unterschleissheim, Germany) following DIN ISO 9277 [13]. The calculation of pore volume and pore diameters followed DIN 66135-2 [14]. Meso- and macropores were calculated via the BET and BJH methods. Sample preparation included a thermal treatment procedure where the samples were degassed for 3 h at 120 °C under reduced pressure using Smart by Micromeritics™ (Unterschleissheim, Germany).

### 2.3. Methods for the Determination of Fresh and Hardened Concrete Properties

The experiments on fresh concrete properties included determinations of bulk density, consistency, and air void content. The investigations of bulk density and air void content were performed in accordance with DIN EN 12350-6 [15] and DIN EN 12350-7 [16]. Due to the high viscosity of HPAC, the procedure to determine the flow spread according to DIN EN 12350-5 [17] had to be modified: The fresh concrete was poured in a truncated cone according to DIN EN 1015-3 [18] (h/d1/d2 = 60/70/100 mm). After striking off the upper surface, the cone was pulled up so that the concrete spread on the table. The diameter of the fresh concrete was measured in two rectangular directions after 30 and 60 s without shocking the table. After 28 days, the compressive strength and the flexural tensile strength were determined by testing 16 × 4 × 4 cm^3^ concrete prisms. The concrete specimens were stored in mixed storage conditions according to DIN EN 12390-2 [19], i.e., the specimens were stripped off the formwork after 1 day and then stored for 7 days under water and 21 days in ambient climate conditions (21 °C ± 1 °C and 50% ± 5% RH). The load speed of the 300 kN strength testing machine (Walter+Bai DB-300-S, Löhningen, Switzerland) was controlled using an electronic device and set to 50 N/s for the flexural tensile strength test and 960 N/s for the compressive strength test by derogation from DIN EN 196-1 [20]. As the very low thermal conductivity of HPAC is one of the main benefits of this building material, special 30 × 30 × 3 cm concrete plates were produced to determine this parameter in a heat flow meter (NETZSCH HFM 436 Lambda, Selb, Germany). For the determination of the shrinking strains, fresh concrete was filled into shrinking drains (l/w/h = 1000/60/40 mm), and the strains were measured for 14 days under a constant temperature of 25.0 °C and a humidity of 40.0%. The strains were measured every minute (f = 0.0167 Hz). Different from other methods, the shrinking drains allow the determination even of deformations resulting from early shrinkage (capillary and chemical shrinkage). For the optical evaluation of the hardened concrete, the specimens were prepared with a hand grinder (ATM Qness, Mammelzen, Germany) using SiC grit-paper with a grain size below 6 µm and investigated with a light microscope (ZEISS Axio Observer Z1 + Software ZEN Core, Oberkochen, Germany).

However, since the aerogels SUFA and SICLA are only produced on a laboratory scale, there were not enough granules to conduct all investigations for each aerogel.

## 3. Results and Discussion

### 3.1. Properties of the Aerogels

The material density of bulk silicon oxide lies in the range of 2.19 g/cm^3^ for amorphous to around 2.65 g/cm^3^ for crystalline (α-quartz) SiO_2_ [21]. For amorphous SiO_2_ aerogels, however, skeletal density is lower and partially dependent on the synthesis route [22,23]. All values shown in Table 3 are in the expected range. The skeletal density of both P100 and P250F are similar, as they just result from the same base material being sieved to obtain different particle size distributions. P100PYR densified during the pyrolysis process. SUFA was the least dense as it stems from hydrophobic precursors, while all other samples were post-functionalized with hydrophobic agents.

Due to the limited information provided by the producers and the limited amounts of aerogel available for proper measurements, only a few envelope and apparent densities were measured.

Through sorption methods, the pore properties seen in Table 4 were measured. The average diameter is roughly in the same region for all samples, with the exception of the densified P100Pyr, SUFA prepared with a hydrophobic precursor, and SICLA Lite with bigger alkoxy groups in its precursor. The last two are a result of repelling or bulky organic groups during synthesis. The surface area shows a bigger range with no clear trend, as it is more prominently influenced by many factors during synthesis. The samples P100 and SICLA > 10% show very similar values, a trend observed throughout this research.

**Figure 2:**
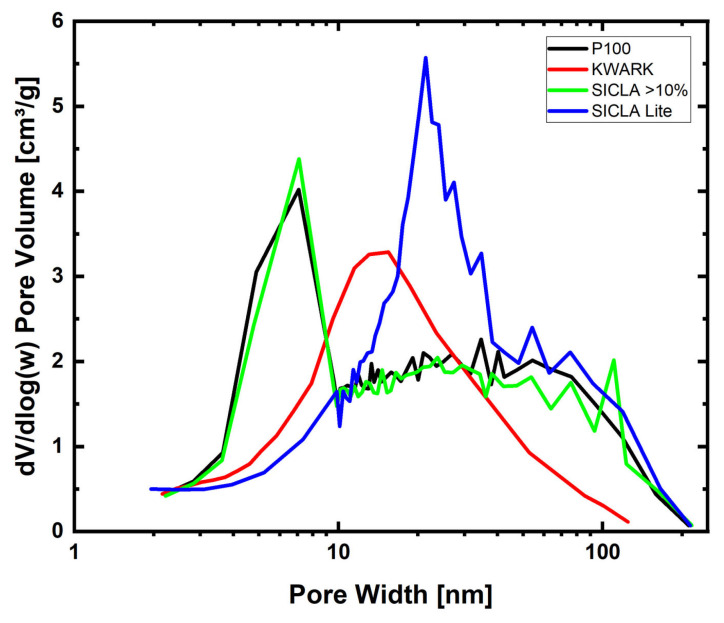
Pore distribution of selected samples in the meso- and lower macroporous regions.

The majority of the mesopores of the samples from Cabot (P100 and P250F) and KEEY (SICLA 3, 5, 10, >10%) are in the range of 7–9 nm, with some examples shown in Figure 2. A majority of Kwark samples are around 10–20 nm, but Kwark samples have a very broad distribution in general, from 8 to 60 nm. SICLA Lite, with a similar broad distribution from 10 to 100 nm, peaks around 20–30 nm. The mesopore distributions of both P100 and P250F are nearly identical to SICLA > 10%, as observed before. All aerogel structures were found to have a monomodal pore size distribution. All of the mentioned ranges are recognizable pore sizes for silica aerogels.

**Figure 3:**
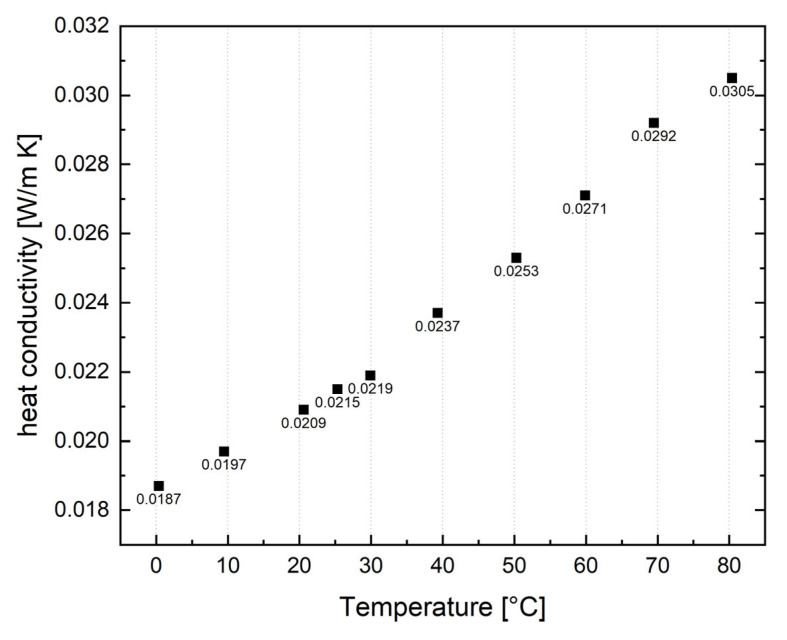
Temperature-dependent heat conductivity, example from sample P100.

Due to the limited amount of aerogel available for proper thermal conductivity measurements, only a few samples could be measured. The thermal conductivity of all samples is very similar and around 0.021 W/(m K), both from the performed measurement and the data provided by the supplier, as aerogels are generally very good thermal insulators. In order to show temperature dependence, P100 was measured accordingly, as seen in Figure 3. Expectedly, higher temperatures lead to a slight increase in thermal conductivity.

**Figure 4:**
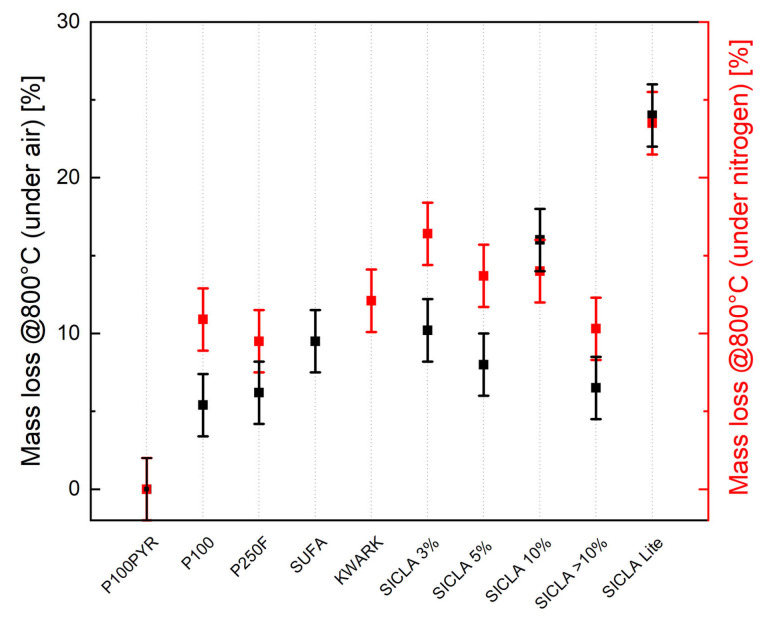
Mass loss of aerogels under air or nitrogen, with SUFA under nitrogen out of range (>70%).

The thermal decomposition behavior, measured via thermogravimetric analysis, is shown in Table 5, with the mass loss also depicted in Figure 4. During the heating process, only the organic part, which is responsible for the hydrophobicity of the material, can burn. Decomposition under nitrogen is a carbonization process that leaves behind a variation of silicon oxycarbide. The more organic content in the original system, the greater the mass loss. Under air, all organic parts are burned, leaving behind a silicon oxide structure. Depending on the number of alkyl groups in the precursors, silicon oxidizes in air and gains additional mass, overall resulting in reduced mass loss compared to decomposition in nitrogen.

With this in mind, the general trends of less mass lost in air and also thermal reactions with oxygen starting at a lower temperature can be explained. Similar to previous data, P100 and SICLA > 10% behave very similarly in terms of thermal decomposition starting points and mass losses. SUFA was revealed to have a significantly higher mass loss, the result of a high alkyl content in the silica precursor instead of added hydrophobic agent. The same phenomena can be observed when performing these experiments on silica–hybrid aerogels.

Detailed discussion of these values without knowing the precise composition of the aerogels and hydrophobic agent would not lead to further information and is therefore omitted in this section. The trends observed, however, still deliver important data for further use of these aerogels in concrete aggregates.

### 3.2. Properties of Fresh Concrete

The properties of fresh concrete are shown in Table 6. The hydrophobicity of each granule type directly affected the required amount of water in each mixture to obtain a consistency comparable to that of HPAC containing Cabot P100. Cabot P100 and Cabot P250F only differ in particle size, so the w/c ratio was kept constant. For the other aerogels, the water content of the concrete mixtures varied from −16% to +133% compared to HPAC_50-P100 (Table 6). The w/c ratio of HPAC_50-P100PYR was significantly higher while retaining a very stiff consistency. This was expected due to the pyrolysis process, which was performed to remove the hydrophobic properties of the granules. The w/c ratio of the concrete containing TIEM SUFA granules was the lowest; nevertheless, the flow spread was higher than that of concrete with P100. The mixtures containing KEEY SICLA and Enersens Kwark indicated relatively high water–cement ratios of 0.304 and 0.358, respectively. As can be seen from Table 6, the flow spread of HPAC_50-P250F was significantly higher than that of HPAC_50-P100, although the w/c ratio was the same (0.243) and could not be decreased due to stiff mixing behavior. The w/c ratio of the mixtures containing SICLA granules varied from 0.243 (HPAC_50-SICLA > 10%) to 0.387 (HPAC_50-SICLA Lite). Within the fresh concrete properties, no relation between the w/c ratio and the consistency was apparent. As mentioned before, not all fresh concrete properties could be determined due to a lack of material.

### 3.3. Properties of Hardened Concrete

**Figure 5:**
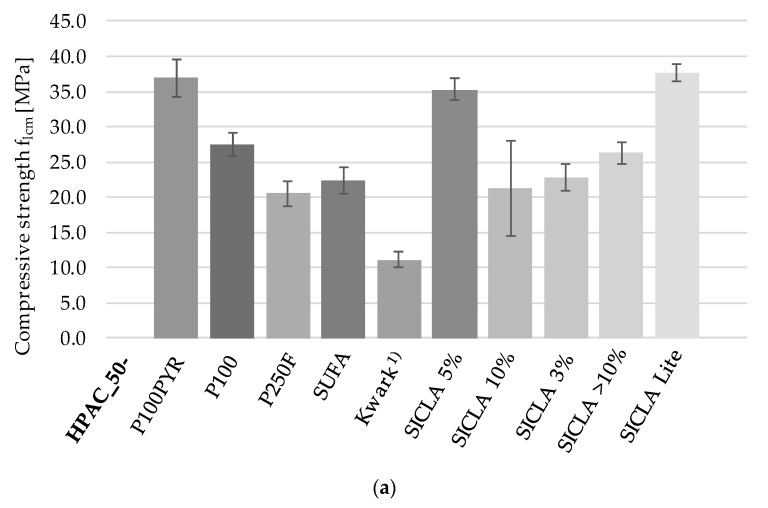

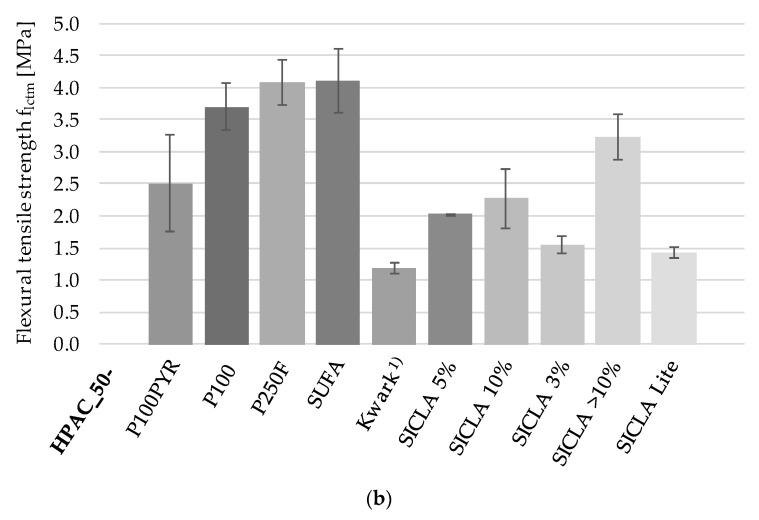
(**a**) Compressive strength and (**b**) flexural bending strength of concrete specimen; average values and standard deviation of at least 3 specimens. ^1)^: tested after 7d.

Figure 5a displays the results of the compressive strength tests. The compressive strength of HPAC_50-SICLA Lite (37.67 MPa) was the highest and even higher than that of HPAC_50-P100 (27.47 MPa), closely followed by HPAC_50-P100PYR with 36.95 MPa and HPAC_50-SICLA 5% with 35.34 MPa. HPAC_50-Kwark specimens showed the lowest compressive strength (11.09 MPa), while the strength of the other specimens ranged from 20.55 MPa to 26.33 MPa.

The observations of flexural tensile strength paint a different picture and are displayed in Figure 5b: The strength of HPAC_50-P250F and HPAC_50-SUFA were comparable (~4.1 MPa) and higher than that of HPAC_50-P100 (3.7 MPa) and HPAC_50-SICLA > 10% (3.23 MPa), while the specimens of the other mixtures showed much lower strengths (1.2 to 2.5 MPa). The specimens with noticeably high compressive strengths, HPAC_50-P100PYR, HPAC_50-SICLA 5% and –SICLA Lite, showed relatively low values for flexural tensile strength. Similarly to its compressive strength values, HPAC_50-Kwark exhibited the lowest value, with 1.18 MPa.

**Figure 6:**
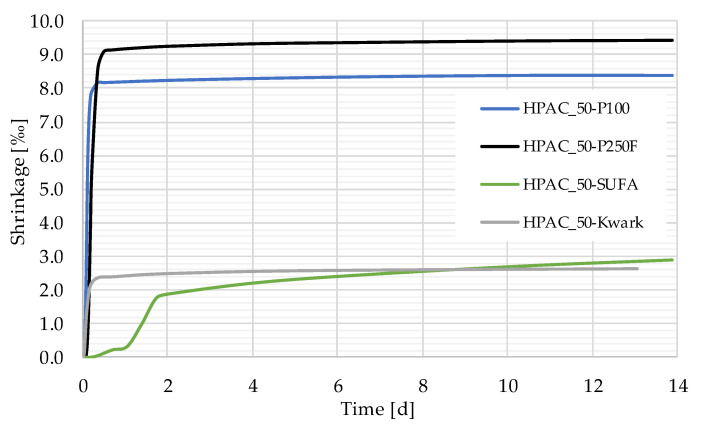
Shrinkage value measured at a temperature of 25.0 °C and a humidity of 40.0%.

Figure 6 shows the shrinkage strains of different concrete mixtures within the first 14 days. The shrinkage strains of HPAC_50-P100, -P250F and -Kwark are similar regarding the shape of their curves, with a steep rise within the first 12 h, followed by a sudden knee and an asymptotical shape until the end of the measurement after 14 d. However, the thresholds of the shrinkage deformations show significant differences, varying between 2.62‰ and 9.44‰. The curve shape for HPAC_50-SUFA is different from the others, showing only a slight rise during the first 24 h, followed by a bigger increase up to the end of the second day and an asymptotic shape to the threshold of 2.87‰. The reference concrete HPAC_50-P100 exhibits a similarly shaped curve to that of -P250F and reaches a shrinkage value of 8.40‰.

The conductivity tests with a heat flow meter yielded single values varying from 0.260 W/mK (P100) to 0.446 W/mK (P100PYR). As already stated in Section 3.1, the provided amount of aerogel was not sufficient to determine the thermal conductivity of every granule type. In Table 7, the average values for λ of every tested HPAC sample are shown.

**Figure 7:**
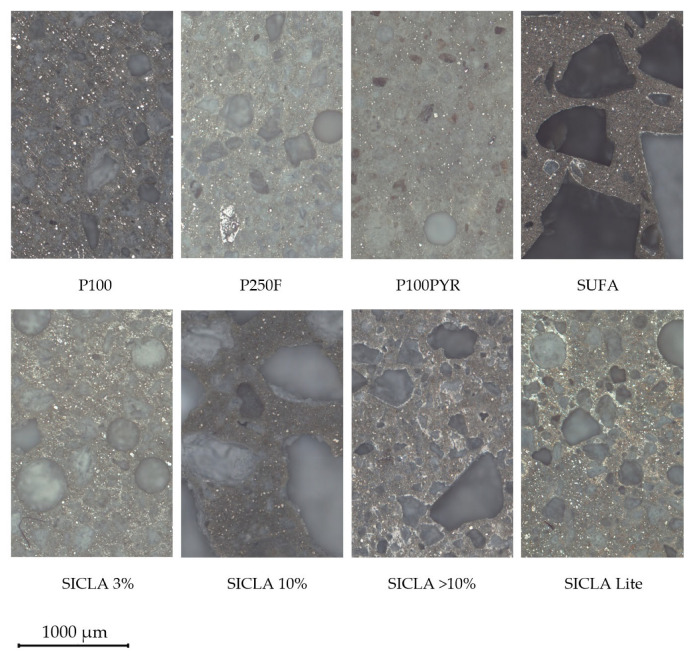
Microscopic images of every HPAC mixture.

In order to evaluate the hardened concrete visually, the samples were investigated with a light microscope (ZEISS Axio Observer Z1, Oberkochen, Germany). This test was primarily conducted to obtain information about the transition zone between the cement matrix and the aerogel particles and the possible resulting influences on concrete strength, as mentioned in Section 1. As can be seen in Figure 7, the concrete specimens differ widely from each other. HPAC_50-SUFA and HPAC_50-SICLA 10% exhibit larger aerogel particles than the other specimens, and the SUFA granule has a striking angular shape. However, this might be due to different grinding/crushing methods used to obtain the particles. The cement matrices of the mixtures showed different shades of grey, which is remarkable since the mixture compositions were the same. No correlation was observed with the water content in the mixture. With regards to the transition zone, it can be seen that the SUFA, SICLA > 10% and SICLA Lite particles are framed in an area of cement paste that is brighter than the regular areas of the matrix. The particles of these mixtures also appear to be embedded with sharper edges than the particles of the other mixtures, which apparently are embedded more homogenously or without any sharp transition. The matrix of P100PYR shows grey streaks around the aerogel particles.

### 3.4. Discussion

**Figure 8:**
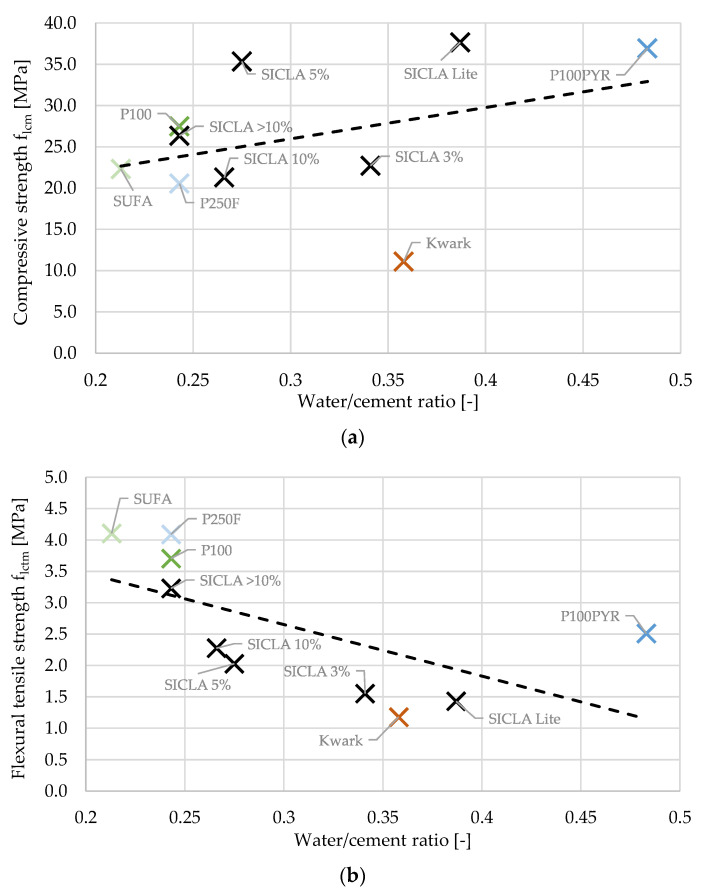
Relation between water–cement ratio and (**a**) compressive strength and (**b**) flexural tensile strength.

A comparison of all HPAC specimens tested in this work regarding the water–cement ratio and concrete strength is shown in Figure 8. Linear correlations were found for both the relations between w/c and compressive strength as well as w/c and flexural tensile strength. While the negative correlation between w/c and flexural strength was expected, because this dependency is known from normal and high-strength concretes, the positive linear correlation of the water–cement ratio and the compressive strength was surprising for the same reason. However, neither the strength of HPAC_50-Kwark for compressive strength nor HPAC_50-P100PYR for flexural tensile strength fit within the general trend. The first might be explained by difficulties finding a flowable mixture and the exchange of the superplasticizer, as already mentioned. For the latter, it might be assumed that there was a kind of inner curing due to the water stored in the hydrophile aerogel particles, preventing the specimens from premature drying and the formation of residual stresses. The grey streaks in the matrix around the particles of P100PYR found in the micrographs might indicate this suction behavior and thus confirm this assumption.

**Figure 9:**
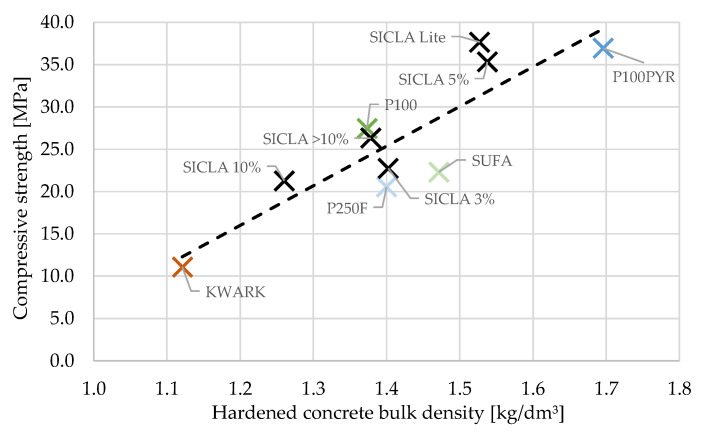
Relation between hardened concrete density and compressive strength.

Opposed to this, the findings on the relation between the density of hardened concrete and compression strength are in agreement with the behavior of HPAC described in the literature [1] and the general dependency of the compressive strength on the density of concretes. As can be seen from Figure 9, a linear correlation was found with R^2^ = 0.76; no relevant trend was observed regarding the relation between the density of hardened concrete and flexural tensile strength.

**Figure 10:**
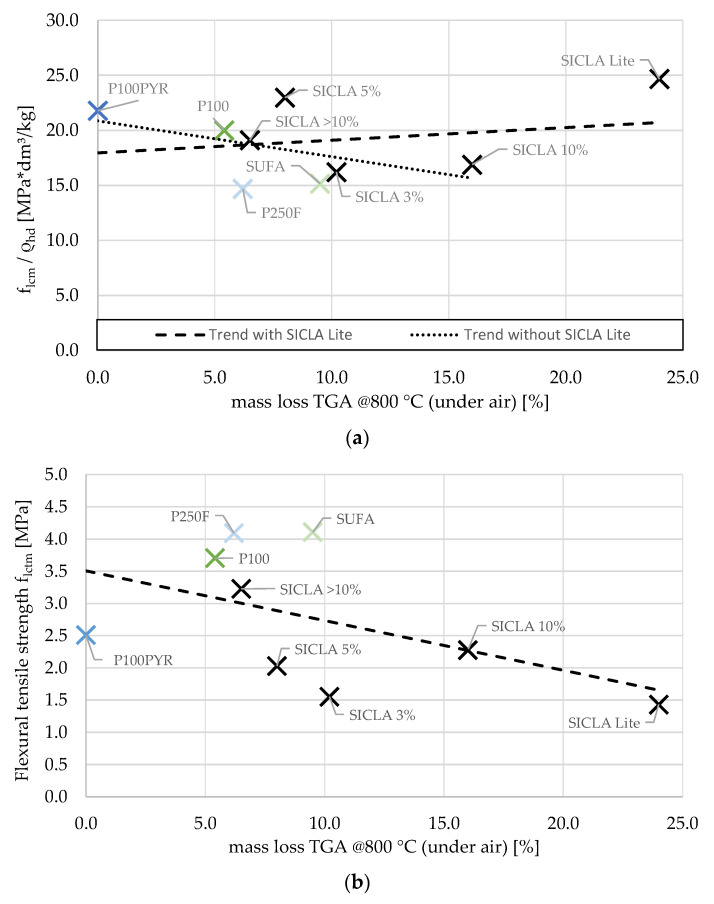
Relation between mass loss in thermogravimetric analysis at 800 °C (under air) and (**a**) compressive strength normalized to density of hardened concrete and (**b**) flexural tensile strength.

To work out possible influences of the aerogel’s hydrophobicity on the strength of the composite, mass losses determined via thermogravimetric analyses were compared with compressive strength values. Because of the aforementioned dependency of the strength on the density of the hardened concrete (Figure 9), this parameter was eliminated by normalization of the strength to the density. From Figure 10a, it is obvious that the extent of the aerogel’s hydrophobicity caused a significant influence on the strength. It appears that the higher the mass loss/hydrophobicity, the higher the compressive strength. Nearly the same result can be obtained when the mass loss is normalized to the BET specific surface area as a measure for the surface wetted with a hydrophobic agent. However, the linear trend shown in Figure 10a is strongly dependent on the result for HPAC_50-SICLA Lite: with its involvement, there is a positive correlation between mass loss and compressive strength. If SICLA Lite is neglected, a trend reversal occurs, implicating that hydrophobicity has a strength-reducing impact. Since the aerogel granule type SICLA Lite was produced with a different precursor (compare Section 2.1), the comparability of the results regarding SICLA Lite should be evaluated carefully. A similar negative trend is found for the relation between mass loss and flexural tensile strength (Figure 10b). These results respond to the expectations concluded from the state of the art: minor hydrophobicity prevents the accumulation of water in the contact zone, resulting in higher strength and minor forming of microcracks. The results of the microscopic investigations might confirm this hypothesis: the nearly white color of the cement matrix in the transition zones of SUFA and SICLA > 10% particles might be an indicator for a higher water or lower cement content in these areas resulting from the higher hydrophobicity of these granules. However, future investigations must explore why HPAC_50-SICLA Lite has the highest compressive strength of all the mixtures despite having a similar appearance in the contact zone. The possible influences of the synthesis method on the properties of the aerogels and the aerogel concrete should also be included in these investigations.

**Figure 11:**
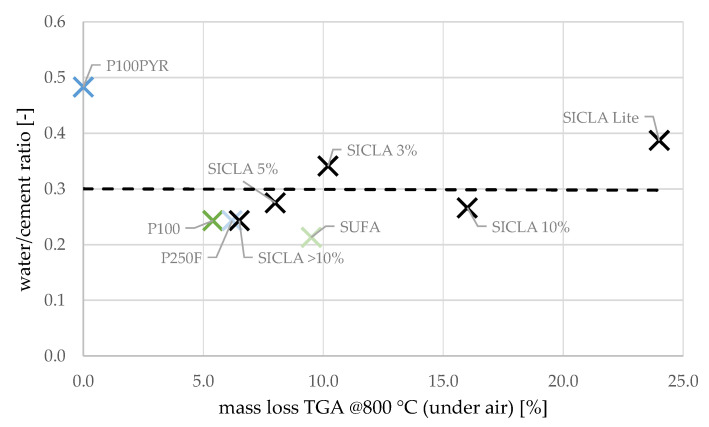
Relation between mass loss in thermogravimetric analysis at 800 °C (under air) and water–cement ratio.

Distinctive trends can be found if the influence of hydrophobicity on water demand is considered (Figure 11). This was not expected, since it was assumed that the hydrophobic surfaces of the aerogel particles would repel the mixing water and thus enhance flowability. In this respect, the granules of HPAC_50-P100PYR respond contrarily to these expectations: by dewatering the mixture due to hydrophile behavior, more water was needed to obtain a flowable mixture. However, it must be emphasized that the water–cement ratio was specified only by visual inspection and was therefore inaccurate. Additional investigations of fresh concrete behavior in relation to flow spread and air void content were only possible to a limited extent due to a lack of material and should be tackled in the future.

As mentioned in Section 1, high-performance aerogel concrete shows some very promising properties that make it interesting for building construction. However, one major disadvantage is its high tendency to shrink. Our own investigations on reinforced construction members made of HPAC revealed that this behavior not only impairs geometrical accuracy, but also causes crack formation due to restrained shrinkage deformations. Up until now, this problem was fixed by adding carbon micro-fibers to the HPAC mixtures [5]. Since this measure involves some new aspects regarding mixing, economy and sustainability, a reduction in shrinkage deformations without the addition of fibers would be a benefit. Against this background, the findings on the shrinkage behavior of HPACs containing different aerogel particles presented in Section 3.3 are very interesting: The investigations of P100 and P250F endorsed the results presented in the state of the art, showing very high shrinkage deformations up to 9.44‰. The shrinkages of the mixtures with SUFA and Kwark were remarkably less, showing deformations up to 70% smaller than those of P100 or P250F, respectively. It can be assumed that the possible reasons for this phenomenon depend on the special properties of each granule: SUFA exhibits a particle compressive strength and compression modulus much higher than all the other granules used in this study (compare Section 2.1). It is conceivable that the particles counteract shrinkage deformation by providing a stiffening grain skeleton. Since the mechanical properties of Kwark are very similar to those of P100, a similar reason for the reduced shrinkage strains of HPAC_50-Kwark is unlikely. As mentioned in Section 2.1, this mixture was the only mixture produced with a different PCE superplasticizer due to a chemical incompatibility of Kwark with the usually used PCE superplasticizer. The additive used instead leads to a higher early strength of concrete, which might affect the development of shrinkage deformations. However, the influence of additives on shrinkage behavior should be investigated in more detail in the future.

**Figure 12:**
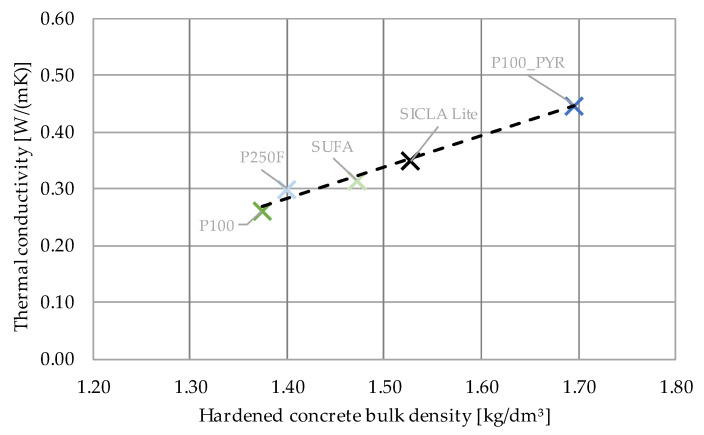
Relation between bulk density and thermal conductivity.

**Figure 13:**
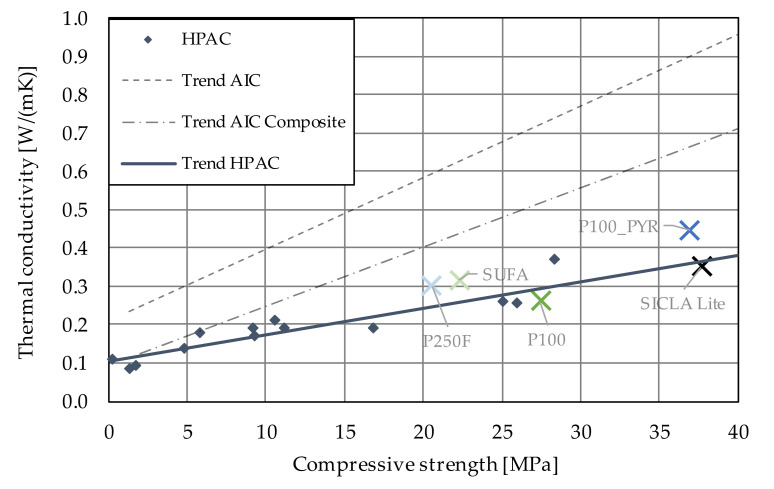
Relation between compressive strength and thermal conductivity.

Because the aerogel content was the same for each mixture, the differences regarding thermal conductivity must depend on the following parameters: (1) The conductivity of the granule itself may influence the conductivity of the composite material. (2) Aerogel granules may be ground while mixing, changing the amount of air voids in the composite as the intact aerogel particles convert to powder. (3) In concrete, air voids naturally occur as a result of the hydration process, where water is bonded chemically and physically. Thus, the water–cement ratio will influence the pore structure. Concretes with a more porous structure tend to have a lower thermal conductivity. It would not be sufficient to refer to the air void content of fresh concrete (see Table 6), as micropores develop during hydration. It can be concluded that parameters (2) and (3) can be revealed by the bulk density of hardened concrete. As shown in Figure 12, thermal conductivity correlates to the bulk density of the specimens. The influences of the different aerogels were not pronounced, which was expected due to the similar thermal conductivities found during the characterization of the granules. In Figure 13, the results of the research presented within this paper are plotted against the trends taken from Figure 1. It becomes obvious that the results fit the trend for all HPAC investigations so far. Thus, all aerogels investigated in this study are suitable for the production of high-performance aerogel concrete. Beyond that, these concretes show an improved thermal insulation performance in comparison with normal concrete (ρ = 2000 kg/m^3^, fck = 8–100 MPa, λ = 1.35 W/mK) [24] or even lightweight concrete with similar properties (ρ = 1100 kg/m^3^, fck = 20–25 MPa, λ = 0.55 W/mK) [25].

## 4. Conclusions

For the determination of fresh and hardened concrete properties, a mixture containing Cabot P100 aerogel, called HPAC_50-P100 (Table 2), served as a reference mixture. Based on this, nine mixtures were produced by substituting different aerogel granules provided by Cabot, TIEM Factory, KEEY aerogels and Enersens for P100 while keeping the amount of aerogel and the other ingredients as constant as possible. A fresh concrete consistency comparable with HPAC_50-P100 was achieved with all aerogel granule types after the water content was adjusted. The water content varied from −16% with TIEM SUFA to +133% by weight with P100PYR compared to the water content of the reference mixture. All granule types allowed for the production of concrete specimens that were suitable for tests of compressive and flexural tensile strength.

According to Table 6, there is no apparent correlation between the water–cement ratio, the consistency, and the air void content, although the differences between the water–cement ratios resulting from the adjustment of the water content were rather high. Likewise, no correlation was found between the hydrophobicity of the aerogels and the water–cement ratio (Figure 11).

The grade of hydrophobicity turned out to have a major influence on the compressive and flexural strength. It was found that the hardened concrete bulk density and the water–cement ratio were in correlation with the compressive strength: while the first correlation for the compressive strength was positive as expected (Figure 9), the latter was surprisingly positive, too (Figure 8a). The highest values were obtained for the mixtures HPAC_50-P100PYR and -SICLA 5% as well as -SICLA Lite, showing compressive strength values even higher than that of the reference mixture with P100. Based on the small amount of data analyzed in this study, it might be concluded that the lower the hydrophobicity, the higher the compressive strength—with the exception of HPAC_50-SICLA Lite, that was produced with a different synthesis routine. So, more detailed investigations on the influence of the precursors and the synthesis method on concrete properties are needed. The measurements of the thermal conductivity of the concrete specimens revealed the sufficiently known correlation between the bulk density of the hardened concrete and the thermal conductivity (Figure 13).

The type of aerogel granule also affected the shrinkage behaviors of the different mixtures: While there was no significant change found for different particle size distributions (P100 and P250F), the influence of the mechanical and chemical properties of the granules was found to be high. The shrinkage deformations measured for HPAC_50-SUFA were ~70% less compared to P100, which might be a consequence of the higher compressive strength and compression modulus of SUFA (Figure 6). HPAC_50-Kwark exhibited low shrinkage deformations in a similar range, although the mechanical properties were different from those of SUFA. However, to obtain workable mixtures with Kwark, the PCE-superplasticizer had to be changed to an additive providing a higher early concrete strength. This potentially affected the development of early shrinkage—an interesting effect that should be studied in more detail in the future.

To investigate the possible effects of the hydrophobicity of the aerogels in the contact zone between aerogel particles and the cement matrix, micrographs were produced (Figure 7). It was found that the contact zone appeared different depending on the granules used. For aerogels with a high hydrophobicity (SUFA, SICLA > 10%), a white boundary was found around the aerogel particles, indicating areas of the matrix low in cement. As mentioned in the state of the art, this might be the origin for the forming of microcracks, resulting in lower compressive strength. However, for HPAC_50-SICLA Lite, no white contact zone was identified, which might be a consequence of the different synthesis routine (Table 1). This should be investigated in more detail by means of scanning electron microscopy.

## Figures and Tables

**Table 1:** Overview of hydrophobic silica aerogel particles and their distributors.

Company & Location	Aerogel Samples
KEEY Aerogel, Habsheim, France	SICLA X% (classic): post-functionalized with different hydrophobic agent content SICLA Lite: different synthesis
Tiem Factory inc., Tokyo, Japan	SUFA: hydrophobic precursor
Cabot Corporation, Frankfurt, Germany	P100 and P250: different size ranges P100PYR: pyrolyzed Cabot P100 sample as unreactive hydrophilic reference
Enersens, Rochetoirin, France	Kwark: post-functionalized

**Table 2:** Composition of the reference mixture HPAC_50-P100 for 1 m^3^ of HPAC.

HPAC_50-P100	Density [kg/dm^3^]	Mass [kg]
CEM I 52.5 R	3.10	798.32
Silica suspension	1.38	207.56
Superplasticizer	1.00	28.33
Water	1.00	90.20
Stabilizer	1.00	3.99
Silica Aerogel Cabot P100	0.098	46.39
Total		1174.8 kg/m^3^

**Table 3:** Available information on/measured densities of the investigated aerogels.

Commercially Available Silica Aerogel Granulate	Skeletal Density [g/cm^3^]	Envelope Density [g/cm^3^]	Apparent Density [g/cm^3^]
P100PYR	2.44	-	-
P100	1.76	0.15	0.09
P250F	1.77	-	0.065–0.085 *
SUFA	1.37	-	-
Kwark	1.63	0.086	0.06
SICLA 3%	1.8	-	0.09
SICLA 5%	1.55	-	0.09–0.105
SICLA 10%	1.6	-	-
SICLA > 10%	1.77	-	0.08
SICLA Lite	1.61	-	0.095–0.1

*: according to Cabot P250F data sheet.

**Table 4:** Pore and thermal properties of the investigated aerogels.

Commercially Available Silica Aerogel Granulate	BJH Average Pore Diameter [nm]	BET Surface Area [m^2^/g]	Thermal Conductivity @25 °C [W/m K]
P100PYR	23.37	763	0.022
P100	12.23	730	0.023
P250F	11.7	704	-
SUFA	14.05	539	-
Kwark	10.28	736	0.021
SICLA 3%	12.42	871	-
SICLA 5%	9.85	925	0.0204 *
SICLA 10%	8.51	992	-
SICLA > 10%	12.1	722	0.021 *
SICLA Lite	19.73	701	0.021 *

*: according to producer KEEY Aerogels.

**Table 5:** Thermal decomposition behavior of the investigated aerogels.

Commercially Available Silica Aerogel Granulate	Beginning of Thermal Decomposition in Air/Nitrogen [°C]	Mass Loss at 800 °C in Air [wt.-%] *	Mass Loss at 800 °C in Nitrogen [wt.-%] *
P100PYR	--/--	~0.0	~0.0
P100	345/395	5.4	10.9
P250F	345/400	6.2	9.5
SUFA	400/375	9.5	71.4–75.7
Kwark	--/375	/	12.1
SICLA 3%	260/335	10.2	16.4
SICLA 5%	265/400	8.0	13.7
SICLA 10%	265/365	16.0	14.0
SICLA > 10%	350/410	6.5	10.3
SICLA Lite	200/475	24.0	23.5

*: measurement error of ± 2.0%.

**Table 6:** Properties of fresh concrete.

HPAC_50-	Water–Cement Ratio ^1^ [-]	Bulk Density [kg/dm^3^]	Flow Spread ^2^ [cm]	Air Void Content [%]
P100PYR	0.483	1.785	- ^3^	3.30
P100	0.243	1.153	25.3/28.0	5.00
P250F	0.243	1.351	37.0/39.0	7.00
SUFA	0.213	1.578	29.3/31.8	4.8
Kwark	0.358	-	-	-
SICLA 3%	0.341	-	-	-
SICLA 5%	0.275	1.361	32.3/36.0	6.80
SICLA 10%	0.266	-	-	-
SICLA > 10%	0.243	-	-	-
SICLA Lite	0.387	-	-	-

^1^: water in silica suspension included. ^2^: average value after 30/60 s. ^3^: not measurable, too stiff.

**Table 7:** Values for the thermal conductivity of HPAC specimens.

HPAC_50-	P100PYR	P100	P250F	SUFA	SICLA Lite
λ [W/mK]	0.446	0.260	0.300	0.315	0.349

## Data Availability

The data presented in this study are available on request from the corresponding author. The data are not publicly available due to privacy issues.
